# Effect of aerobic exercise on brain metabolite profiles in the mouse models of methamphetamine addiction: LC-MS-based metabolomics study

**DOI:** 10.1186/s12888-023-05351-1

**Published:** 2023-11-17

**Authors:** Jisheng Xu, Zhicheng Zhu, Yu Jin, Changling Wei, Yi Wang, Xue Li

**Affiliations:** 1 School of Sports Medicine and Health, Chengdu Sport University, Chengdu, 610041 P. R. China; 2 Department of Epidemiology and Population Health, Albert Einstein College of Medicine, Bronx, NY 10461 USA

**Keywords:** Methamphetamine, Aerobic exercise, Metabolomics, Brain, Substance abuse

## Abstract

Methamphetamine (MA) abuse is recognized as a brain disorder, and physical activity has clear benefits for MA use disorders. The specific mechanisms by which physical activity alleviates MA use disorders are currently not fully understood. Based on this, the present study used untargeted metabolomics using liquid chromatography-mass spectrometry (LC-MS) to analyze the metabolic changes induced by MA in the brains of mice by exercise intervention. It was found that after 2 weeks of treadmill training, aerobic exercise modulated MA-induced brain metabolic disorders, in which 129 metabolites existed that were significantly differentiated in response to MA induction, and 32 metabolites were significantly affected by exercise. These differential metabolites were mainly enriched in glycerophospholipid metabolism, steroid hormone biosynthesis and degradation, and renin-angiotensin system pathways. To our knowledge, this study is the first to use LC-MS to investigate the effects of aerobic exercise on MA-induced brain metabolic profiling. The findings of this study provide new insights into exercise therapy using MA.

**Supplementary Information:**

The online version contains supplementary material available at 10.1186/s12888-023-05351-1.

## Introduction

Methamphetamine (MA) abuse is a global public health problem [1, 2], causing serious medical burden and social impact [3, 4]. MA is a highly addictive, widely abused amphetamine-type psychostimulant [1]. Its high lipid solubility enables it to cross the blood-brain barrier (BBB) and reach the brain rapidly [5], causing neurotoxicities such as high temperature, neuroinflammation, and oxidative stress, resulting in brain and central nervous system damage [6–9]. Based on trials, MA abuse can lead to reactions such as hypertension, emotional agitation, behavioral disinhibition, and anxiety in individuals [10–13]. This condition is accompanied with extremely complex and intense withdrawal symptoms [14], such as anhedonia [15], aggression [16], sleep disturbance [17], and strong cravings after drug discontinuation [18]. Addiction-related symptoms and neurological damage are caused by drug-induced metabolic disturbances [19].

Physical activity is widely considered an intervention for the treatment of substance use disorders [20]. Clinical studies indicated that regular exercise can help MA-dependent patients reduce drug cravings [21], and improve physical, mental, and social health [22, 23]. Exercise can also improve the brain function of MA-dependent patients, such as improving cognitive impairment [24] and strengthening inhibitory control functions [25]. Moreover, exercise can alleviate MA-induced neurotoxicity [26], stabilize BBB integrity [27], and improve MA-induced reductions in striatal dopaminergic markers [28]. Although exercise improves the symptoms and mechanisms of MA dependence, the mechanism in which exercise modulates MA-induced metabolic homeostasis in the brain remains unclear. Metabolomics has the advantages of high sensitivity and specificity [29] and can discover potential biomarkers by studying the perturbation of metabolic state, which can help in revealing the underlying mechanism of diseases [19, 30]. Currently, this emerging method can be used to identify changes in metabolites affected by addictive drugs [19]. Clinical studies have found that compared with non-drug users, MA abusers have disturbances in serum metabolism, including phenylalanine metabolism, glyoxylate and dicarboxylate metabolism, and alanine, aspartate and glutamate metabolism, which may partially explain the oxidative stress and neuroinflammatory changes induced by MA [31]. Furthermore, metabolomics studies based on magnetic resonance imaging and positron emission tomography suggested that brain volume is affected in MA-dependent individuals, and the major metabolites in brain tissue are abnormally represented, including N-acetylasparate and total creatine [32]. Preclinical experiments have also provided evidence for metabolic disorders in MA addiction. MA-administered rats exhibited rapid and marked changes in energy metabolism, nervous system, and membrane lipid metabolism [33, 34], inducing neurotoxicity and locomotor sensitization, and depression-, and anxiety-like behaviors [35]. Human brain tissue and cerebrospinal fluid are not readily available because of ethical and safety constraints [36]. Although the brain tissue can be selected for metabolomic analysis in animal models, the current evidence for brain metabolomic studies on MA administration is still limited [37].

Conditioned place preference(CPP) reflects an acquired associative memory model linking drug rewards to neutral environmental cues [38]. Animals selectively move freely between the two environments, using the time they spend in drug-related environments as an indicator of drug preference [39, 40]. Previous studies have found that CPP-validated MA addiction model mice cause changes in multiple brain regions, such as the nucleus accumbens [41], hippocampus [42], and hypothalamus [43], and are associated with changes in the trends of addiction-related neurotransmitters such as dopamine and glutamate [44, 45]. Based on this, we hypothesize that exercise can modulate the metabolic homeostasis of the MA-induced brain. In this study, MA-induced metabolome in mouse brain tissue was investigated based on liquid chromatography-tandem mass spectrometry (LC-MS) and the metabolite profiles of exercise and non-exercise MA-treated mice were compared. These biomarkers and their enrichment pathways may explain the changes in brain function in MA addiction, and provide new insights into the field of exercise therapy for MA addiction.

## Methods

### Animals

The male C57BL/6J mice (8-week-old, body weight 18–22 g) used in this study were obtaineded from Chengdu Dashuo Experimental Animals Co. Ltd. (Chengdu, China) and bred in the animal laboratory of Chengdu Sport University. All mice were allowed to feed and water ad libitum, ensured a 12-hour light/dark cycle, a temperature of 22 ± 2 °C, and humidity of 52 ± 2%, and were reared adaptively for 1 week. All experimental procedures were approved by the Academic Committee of Chengdu Sport University (No: 2022-56) following the National Guidelines for the Care and Use of Laboratory Animals.

### Experimental designs

**Fig. 1:**
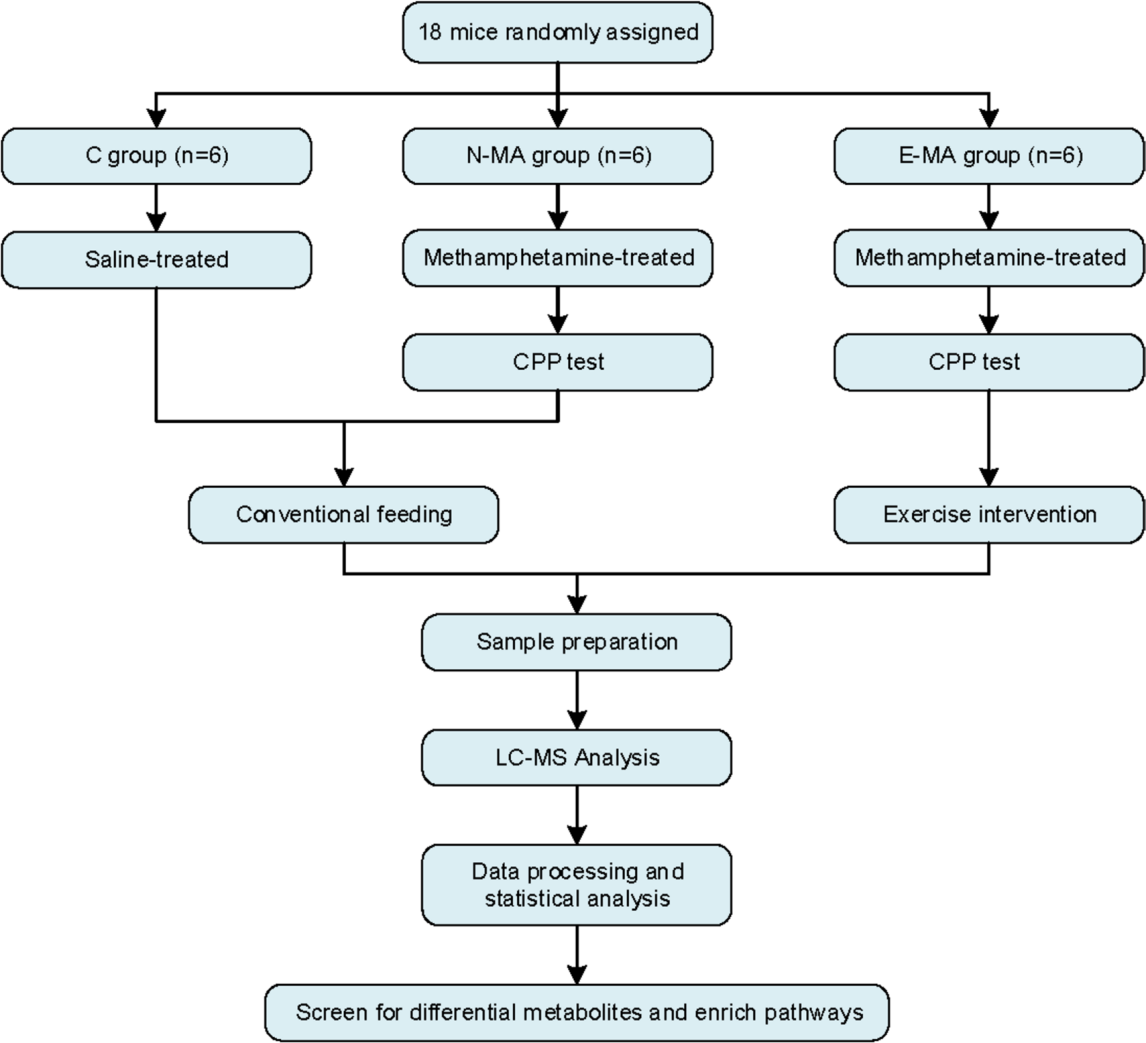
Experimental flowchart

**Fig. 2:**
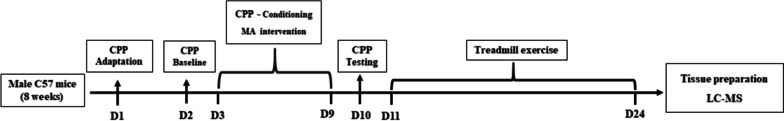
Experimental schedules chart

Eighteen mice were randomly assigned to the control group (C group, *n* = 6), methamphetamine control group (N-MA group, *n* = 6), and methamphetamine exercise group (E-MA group, *n* = 6). The mice in group C were injected with normal saline, and group N-MA and E-MA were modeled for MA addiction as previously described [46]. MA was obtained from the Sichuan Key Laboratory of Intelligent Police, Sichuan Police College (Luzhou, China), and dissolved in 0.9% NaCl (saline). Mice received intraperitoneal injection of MA (1 mg/kg) or an equal volume of 0.9% saline at 8 am every day for seven consecutive days, and the model was validated by CPP. Afterward, the mice in the C group and N-MA group were kept in cages for 2 weeks without any drug or any intervention. The mice in the E-MA group were exercised on a treadmill (SA101, SANS, Jiangsu, China). The exercise protocol was based on previous studies and modified according to actual conditions [47]. Briefly, exercise training was carried out at moderate intensity (12 m/min, 1 h per day) for 2 weeks When the mice stopped exercising, continued exercise was ensured by gently touching their tail). After the two-week exercise intervention, all experiments were conducted strictly in accordance with the Guidelines for the Care and Use of Laboratory Animals (GB/T 35,892 − 2018) to ensure ethical review. All experimental animals were humanely euthanized using a standardized procedure. Specifically, the animals were first anesthetized with an intraperitoneal injection of 0.5% sodium pentobarbital and then euthanized by cervical dislocation to ensure they were fully anesthetized and minimize their pain and suffering. All operators were fully trained and made every effort to handle the animals with care and compassion. After dissection according to the brain atlas, the brain (excluding the cerebellum) was excised and immediately placed in liquid nitrogen for the preparation of subsequent analysis. All experiments were conducted at the Sichuan Provincial Key Laboratory of Sports Medicine, Chengdu Sport University The experimental flow is presented in Fig. 1 and the experimental schedule is shown in Fig. 2.

### CPP test

The experimental procedures for CPP are based on previous reports with minor modifications [43, 48]. The CPP apparatus was obtained from Jiangsu SANS Company (Jiangsu, China), including two compartments of the same size on the left (black striped wall) and right (white wall) (15 cm × 17 cm × 20 cm) and a central corridor (6 cm × 15 cm × 20 cm). A camera was placed on top of each compartment, and all data were recorded using a computerized video tracking system. The experimental process involved an unbiased apparatus and a balancing procedure, which consisted of four phases as follows:

Adaptation phase (Day 1): Mice were placed from the central corridor and allowed to walk freely in the CPP apparatus for 10 min, to ensure that they were acclimated to the environment.

Baseline phase (Day 2): Each mouse was placed in the central hallway and allowed to explore freely in both compartments for 10 min, and the time spent in each compartment was recorded. This method aimed to determine whether the mice have an intrinsic preference and to identify drug-related compartments.

Conditioning phase (Day 3–9): First, the mice in the model group were injected with methamphetamine (1 mg/kg), and then placed in the drug-related compartment (assumed to be the right compartment) for 30 min. Control experiments were performed 8 h later on the same day. Experimental animals were injected with saline and restrained in a non-medicated compartment for 30 min. To ensure the balance of the experiment, we injected the saline control group with an equal volume of saline.

Testing phase (Day 10): Drug injection was stopped and mice were placed in the central corridor and allowed to freely explore both compartments for 10 min. Then, the time spent in each compartment was recorded. The preference score was defined as the time spent in the drug-associated compartment during the test phase minus the time spent in the drug-associated compartment during the baseline phase [49].

### Metabolite profiling

LC-MS analysis was carried out using Dionex U3000 ultra-high performance liquid chromatography-tandem Q exactive plus high-resolution mass spectrometer system (Thermo Fisher Scientific, Waltham, MA, USA). All solvents and chemicals were of analytical or chromatographic grade. Water, methanol, acetonitrile, and formic acid were obtained from Thermo Fisher Scientific. L-2-Chlorophenylalanine was obtained from Shanghai HC Biotechnology Co., Ltd. (Shanghai, China). Samples were processed in the following steps. Samples were thawed in an ice bath to minimize degradation. Tissue samples were accurately weighed (30 mg) and transferred to a 1.5 ml Eppendorf tube. Approximately 20 µL of L-2 chlorophenylalanine (0.06 mg/ml) was dissolved in methanol as an internal standard, and then added with 400 µL of a mixture of methanol and water (4/1, vol/vol). After the samples were pre-cooled at − 20 °C in a refrigerator for 2 min, the samples were crushed in a grinder (60 Hz, 2 min). The whole sample was extracted in an ice-water bath for 10 min, followed by standing at -20 °C for 30 min. The extract was centrifuged for 10 min (13,000 rpm, 4 °C), and 300 µL of the supernatant was loaded into an LC-MS injection vial to evaporate the sample. After ward, 300 µL of methanol-water (1/4, vol/vol) was reconstituted (vortex for 30 s, sonicated for 3 min) and allowed to stand at − 20 ℃ for 2 h. The samples were then centrifuged for 10 min (13,000 rpm, 4 °C), pipetted with a syringe with 150 µL of the supernatant, filtered with a 0.22 μm organic-phase pinhole filter, transferred to an LC injection vial, and stored at − 80 °C until LC-MS analysis was performed. Quality control samples were prepared by mixing equal volumes of extracts from all samples. Detailed information in Supplementary Material S1.

### Statistical analysis

CPP data were statistically analyzed using SPSS 26.0 (IBM SPSS Statistics) and graphed using GraphPad PRISM 9.0. The place preference of mice in the baseline and test phases for the left and right compartments and the place preferences of the two groups of mice in the test phase E-MA and N-MA were compared using independent samples t-tests. Results are expressed as mean ± standard, and *p*<0.05 is considered statistically significant.

The LC-MS raw data were subjected to baseline filtering, peak identification, integration, retention time correction, peak alignment, and normalization by using metabolomics processing software Progenesis QI v2.3 (Nonlinear Dynamics, Newcastle, UK). Univariate analysis and multivariate analysis were combined to identify the differential metabolites. Multivariate statistical analysis was used to distinguish the overall differences in metabolic profiles between groups and determine differential metabolites between groups. Unsupervised principal component analysis (PCA) was used to observe the overall distribution between samples and the stability of the entire analysis process. The orthogonal partial least squares discriminant analysis (OPLS-DA) can maximize the difference between different groups within the model.

In addition, 200- response permutation testing(RPT) was used to examine the model quality. In the RPT test, the established OPLS-DA model is considered reliable if the intercept of the Q2 regression line was negative. Variable important in projection (VIP) was determined according to the OPLS-DA model. Univariate statistical analysis was performed using Student’s t-test for data with parametric distributions to compare metabolites between the two groups. Among these metabolites, metabolites with *p* < 0.05 in the T-test were considered as differentially expressed metabolites. Accordingly, metabolites with VIP > 1 in OPLS-DA were considered potential biomarkers. Kyoto encyclopedia of genes and genomes (KEGG) [50] analysis was used to identify potentially deranged metabolic pathways (http://www.genome.jp/kegg/).

## Results

### CPP result

**Fig. 3:**
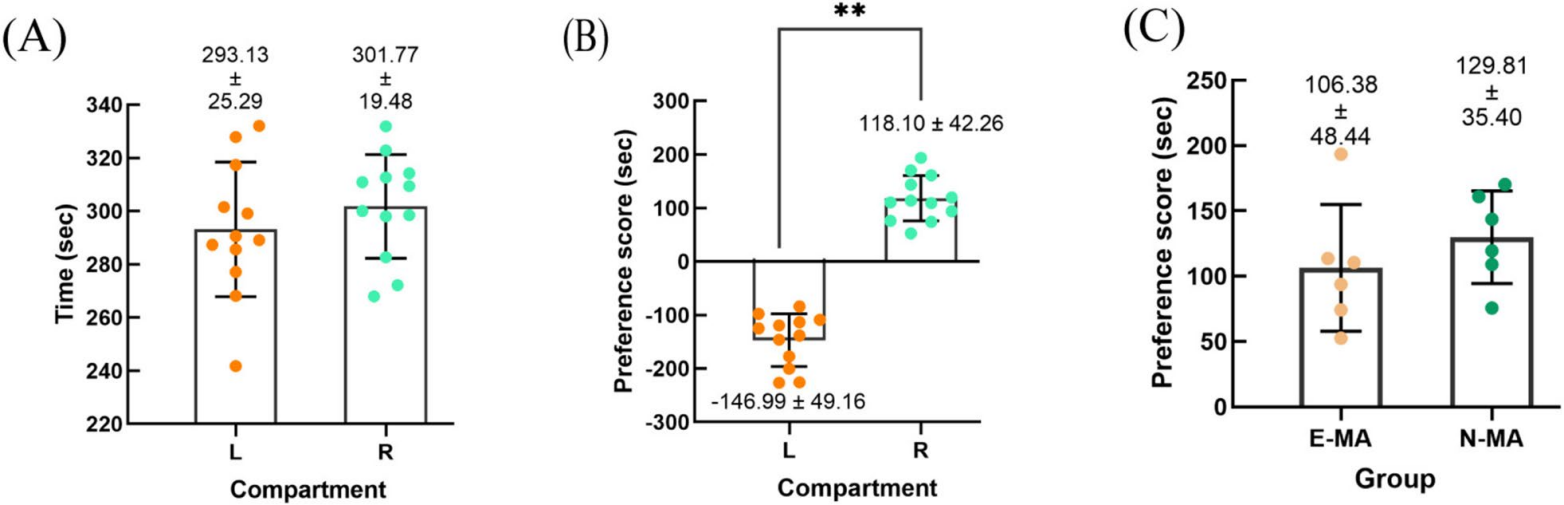
Methamphetamine (MA)-induced CPP in mice. **A** Baseline phase: Mice have no intrinsic preference for L and R compartments. **B** Testing phase: Mice produce place preference for MA administration compartment (R). **C** There was no statistical difference in the place preference of the MA administration compartment (R) between mice in the E-MA and N-MA group. L: left compartment.; R: right compartment; ** P <0.01, A highly significant statistical difference was observed

The CPP data showed no statistical difference in the time spent by the mice in the left and right compartments during the baseline phase (*P* = 0.36), thus excluding an intrinsic location preference. The right compartment was identified as the target compartment for MA administration. The results of the test phase showed that after MA adjustment, the mice’s place preference scores in the right compartment (119.10 ± 42.26) were significantly higher than those in the left compartment (–146.99 ± 49.16) after drug treatment was stopped (*P*<0.01), indicating that the modeling of MA dependence was successful. No significant difference in the CPP scores was observed in the right compartment between the mice randomly assigned to the E-MA group and the N-MA group (*P* = 0.36 Fig. 3).

### MA-induced metabolic profile changes

**Fig. 4:**
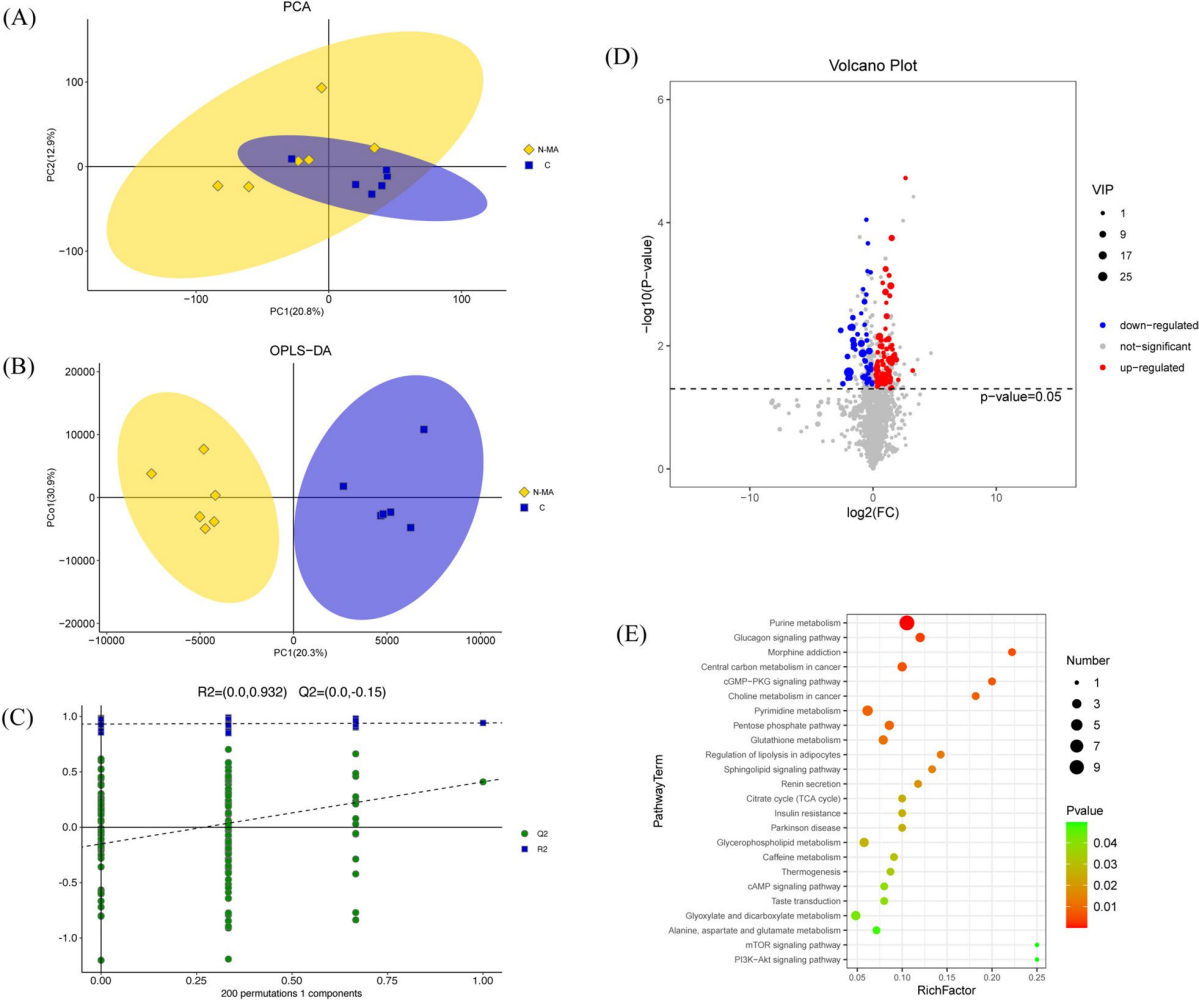
Differences in metabolic profiles between the C and N-MA group. **A** PCA score plot. **B** OPLS-DA score plot. **C** 200- response permutation testing chart for OPLS-DA. **D** Volcano plots of differentially abundant metabolites. **E** Bubble chart of metabolic pathways affected by methamphetamine from KEGG enrichment

After preprocessing and normalizing the data, 8,864 metabolic signatures were subjected to statistical analysis. PCA1 in the PCA plot showed significant differences in metabolic characteristics between the N-MA and C group (Fig. 4A). Afterward, OPLS-DA was used to clearly explain the differences between the two groups (Fig. 4B). The RPT results of R2=(0.0, 0.932), Q2=(0.0, − 0.15) ensure the reliability of the model (Fig. 4C).

A search was initiated for potential metabolites responsible for the differences between groups. The thresholds were set at VIP>1 and *p*<0.05, and a total of 129 differential metabolites were identified (Table 1). In comparison with the C group, 80 metabolites were substantially increased in the N-MA group (red dots), and 49 metabolites were significantly decreased (blue dots, Fig. 4D). The enrichment analysis of 129 different metabolites was carried out through the KEGG database, and 24 pathways were obtained (*P*<0.05) (Fig. 4E), including glucagon signaling pathway, sphingolipid signaling pathway, renin secretion, glycerophospholipid metabolism, and alanine, aspartate, and glutamate metabolism.

**Table 1:** Differential metabolites screened from the N-MA and control groups

NO.	Metabolite	*p* Value	VIP	Fold Change	Variations versus controls
1	Adenosine	0.027	29.103	0.256	↓
2	Psilocybin	0.013	16.409	0.557	↓
3	PE(0:0/22:6(4Z,7Z,10Z,13Z,16Z,19Z))	0.017	15.318	2.575	↑
4	LysoPC(0:0/16:0)	0.038	14.020	1.708	↑
5	1-Pentanesulfenothioic acid	0.007	11.427	1.444	↑
6	PC(18:1(11Z)/0:0)	0.030	11.171	1.903	↑
7	Adenosine 3’-monophosphate	0.005	11.133	0.308	↓
8	Oxidized glutathione	0.009	10.776	0.515	↓
9	PS(22:6(4Z,7Z,10Z,13Z,16Z,19Z)/0:0)	0.001	10.690	2.711	↑
10	Mahaleboside	0.032	10.226	0.619	↓
11	LysoPE(22:6(4Z,7Z,10Z,13Z,16Z,19Z)/0:0)	0.017	9.542	2.969	↑
12	Pyrrolidonecarboxylic acid	0.012	9.494	0.807	↓
13	LysoPC(22:6(4Z,7Z,10Z,13Z,16Z,19Z)/0:0)	0.015	8.706	3.258	↑
14	Prostaglandin E2 p-benzamidophenyl ester	0.001	8.629	2.017	↑
15	Adenosine monophosphate	0.005	8.497	0.285	↓
16	LysoPC(20:4(8Z,11Z,14Z,17Z)/0:0)	0.018	8.454	2.647	↑
17	Choline	0.024	8.366	1.230	↑
18	LysoPE(0:0/20:4(8Z,11Z,14Z,17Z))	0.037	8.329	2.085	↑
19	Guanidylic acid (guanosine monophosphate)	0.010	8.045	0.341	↓
20	Guanosine 3’-monophosphate	0.008	8.034	0.326	↓
21	Hypoxanthine	0.038	7.531	1.260	↑
22	Citric acid	0.022	7.061	1.262	↑
23	p-hydroxyfosinoprilat	0.000	6.772	2.858	↑
24	Pyridine N-oxide glucuronide	0.008	6.549	2.377	↑
25	Isocitrate	0.029	5.888	1.214	↑
26	Calcimycin	0.003	5.743	2.159	↑
27	PI(20:4(5Z,8Z,11Z,14Z)/0:0)	0.001	5.657	2.036	↑
28	L-Carnitine	0.024	5.438	0.791	↓
29	LysoPE(20:4(5Z,8Z,11Z,14Z)/0:0)	0.026	5.226	2.469	↑
30	PE(18:1(9Z)/0:0)	0.040	5.176	1.601	↑
31	Uridine diphosphate-N-acetylglucosamine	0.002	4.957	0.619	↓
32	LysoPE(18:0/0:0)	0.047	4.775	1.560	↑
33	Adenylsuccinic acid	0.006	4.678	0.162	↓
34	PC(18:1(9Z)/0:0)	0.041	4.646	1.979	↑
35	Benthiavalicarb isopropyl	0.015	4.526	0.237	↓
36	D-Gluconic acid Mn(II) salt	0.003	4.419	0.321	↓
37	FAPy-adenine	0.033	4.407	0.273	↓
38	L-Acetylcarnitine	0.018	4.380	0.645	↓
39	Dehydroepiandrosterone (DHEA)	0.036	4.126	2.597	↑
40	N2,N2-Dimethylguanosine	0.041	4.095	0.183	↓
41	(R)-(+)-2-Pyrrolidone-5-carboxylic acid	0.010	4.061	1.510	↑
42	PC(22:6(4Z,7Z,10Z,13Z,16Z,19Z)/0:0)	0.017	4.034	3.717	↑
43	Glucose-uridine-C1,5’-diphosphate	0.032	4.014	0.569	↓
44	PC(20:4(5Z,8Z,11Z,14Z)/0:0)	0.019	3.934	2.890	↑
45	Glycolic acid	0.020	3.474	1.766	↑
46	Xanthine	0.008	3.417	1.568	↑
47	3-Methyl sulfolene	0.007	3.390	1.344	↑
48	p-CHLOROPHENYLALANINE	0.040	3.340	0.937	↓
49	PG(18:3(6Z,9Z,12Z)/0:0)	0.023	3.061	2.151	↑
50	4’-Demethyldeoxypodophyllotoxin	0.029	2.963	1.687	↑
51	LysoPE(18:1(9Z)/0:0)	0.043	2.824	1.645	↑
52	Uridine	0.046	2.789	1.279	↑
53	5-Hydroxy-N-formylkynurenine	0.031	2.707	0.246	↓
54	PHODiA-PA	0.002	2.616	2.569	↑
55	2,3,4-Trihydroxybutanoic acid	0.045	2.557	1.498	↑
56	PS-PE	0.001	2.478	2.476	↑
57	1-(2-methoxy-6Z-heptadecenyl)-sn-glycero-3-phosphoethanolamine	0.047	2.440	1.594	↑
58	Tiocarbazil	0.046	2.331	1.642	↑
59	Norepinephrine sulfate	0.011	2.311	2.146	↑
60	Metam-sodium	0.035	2.290	0.708	↓
61	C16 Sphinganine	0.023	2.262	0.871	↓
62	DL-Acetylcarnitine	0.040	2.197	0.706	↓
63	2,4-Diamino-6,7-dimethoxyquinazoline	0.018	2.188	1.305	↑
64	PC(22:4(7Z,10Z,13Z,16Z)/0:0)	0.014	2.082	3.391	↑
65	Dimethoate	0.034	2.071	0.239	↓
66	PC(2:0/1:0)[U]	0.049	2.028	2.656	↑
67	LysoPI(16:0/0:0)	0.030	2.009	2.483	↑
68	Phosphoribosyl-AMP	0.006	2.000	0.419	↓
69	Uracil	0.013	1.998	1.304	↑
70	Ribose 1-phosphate	0.020	1.986	0.886	↓
71	Thioperamide	0.035	1.962	2.071	↑
72	Thiabendazole	0.042	1.931	0.939	↓
73	Adenosine 3’,5’-diphosphate	0.008	1.876	0.707	↓
74	PIM1(17:0/18:1(9Z))	0.025	1.861	9.323	↑
75	LysoPC(16:1(9Z))	0.011	1.826	1.653	↑
76	PC(24:0/P-18:1(11Z))	0.024	1.822	0.869	↓
77	LysoPC(20:3(5Z,8Z,11Z)/0:0)	0.011	1.818	2.985	↑
78	D-Tagatose 1,6-bisphosphate	0.015	1.788	1.877	↑
79	Uridine monophosphate (UMP)	0.011	1.780	0.324	↓
80	Isopropalin	0.036	1.735	1.797	↑
81	3b,16a-Dihydroxyandrostenone sulfate	0.048	1.696	2.890	↑
82	MG(0:0/20:4(5Z,8Z,11Z,14Z)/0:0)	0.001	1.655	1.722	↑
83	Succinyladenosine	0.036	1.651	0.781	↓
84	Caffeic acid 3-sulfate	0.008	1.638	2.054	↑
85	5-(2’-Carboxyethyl)-4,6-Dihydroxypicolinate	0.020	1.609	1.248	↑
86	Aminoparathion	0.040	1.606	1.417	↑
87	1-Oleoylglycerophosphoserine	0.010	1.567	2.641	↑
88	Deoxyguanosine diphosphate (dGDP)	0.001	1.559	0.685	↓
89	LysoPC(18:2(9Z,12Z))	0.043	1.544	1.803	↑
90	Diethyl disulfide	0.000	1.413	0.749	↓
91	Retinylphosphate mannose	0.013	1.387	1.799	↑
92	FAD	0.001	1.384	0.875	↓
93	Tetracenomycin D1	0.010	1.372	2.931	↑
94	5’-CMP	0.000	1.370	0.687	↓
95	LysoPE(0:0/18:2(9Z,12Z))	0.025	1.357	2.291	↑
96	GDP-L-fucose	0.007	1.341	0.689	↓
97	Isosorbide Dinitrate	0.033	1.332	1.487	↑
98	Tazobactam	0.039	1.322	2.204	↑
99	Tetrahydrodipicolinate	0.005	1.309	0.626	↓
100	Dibutyl sulfide	0.021	1.307	1.322	↑
101	Hirsutin	0.000	1.291	6.223	↑
102	PG(22:6(4Z,7Z,10Z,13Z,16Z,19Z)/0:0)	0.015	1.277	2.922	↑
103	Suprofen	0.043	1.269	1.442	↑
104	Sphinganine	0.025	1.239	0.894	↓
105	5β-Chola-3,8(14),11-trien-24-oic Acid	0.036	1.239	4.146	↑
106	alpha-D-Glucose 1,6-bisphosphate	0.010	1.217	1.679	↑
107	Ethyl 3-mercaptobutyrate	0.034	1.216	0.813	↓
108	7b-Hydroxy-3-oxo-5b-cholanoic acid	0.038	1.215	2.328	↑
109	2,7-Anhydro-alpha-N-acetylneuraminic acid	0.032	1.205	1.206	↑
110	Imidazoleacetic acid riboside	0.022	1.201	0.693	↓
111	N-(2,3-Dihydroxybenzoyl)-L-serine	0.017	1.193	1.657	↑
112	Myristic acid	0.028	1.189	0.758	↓
113	PE(18:2(9Z,12Z)/0:0)	0.033	1.186	2.724	↑
114	gamma-Glutamylalanine	0.035	1.148	0.776	↓
115	D-Glycerate 3-phosphate	0.029	1.108	2.097	↑
116	Penciclovir	0.039	1.099	1.576	↑
117	N-Acetyl-b-neuraminic acid	0.017	1.098	0.611	↓
118	CDP-ethanolamine	0.047	1.080	0.674	↓
119	DPA	0.039	1.066	2.617	↑
120	8,11,14-Eicosatrienoic acid	0.024	1.063	1.122	↑
121	Mangold’s acid	0.026	1.056	1.373	↑
122	2-Hydroxy-6-ketononatrienedioate	0.035	1.053	0.719	↓
123	1-O-(2R-methoxy-hexadecyl)-sn-glycerol	0.002	1.047	2.111	↑
124	SULFAMONOMETHOXINE	0.001	1.045	0.729	↓
125	Xanthosine	0.005	1.030	2.011	↑
126	1-Acetoxy-2-hydroxy-16-heptadecen-4-one	0.001	1.027	0.574	↓
127	PI(22:6(4Z,7Z,10Z,13Z,16Z,19Z)/0:0)	0.003	1.021	0.514	↓
128	D-Erythrose 4-phosphate	0.040	1.016	1.271	↑
129	2-Hydroxyadenine	0.012	1.003	0.374	↓

### Exercise regulates MA-induced metabolic signatures

**Fig. 5:**
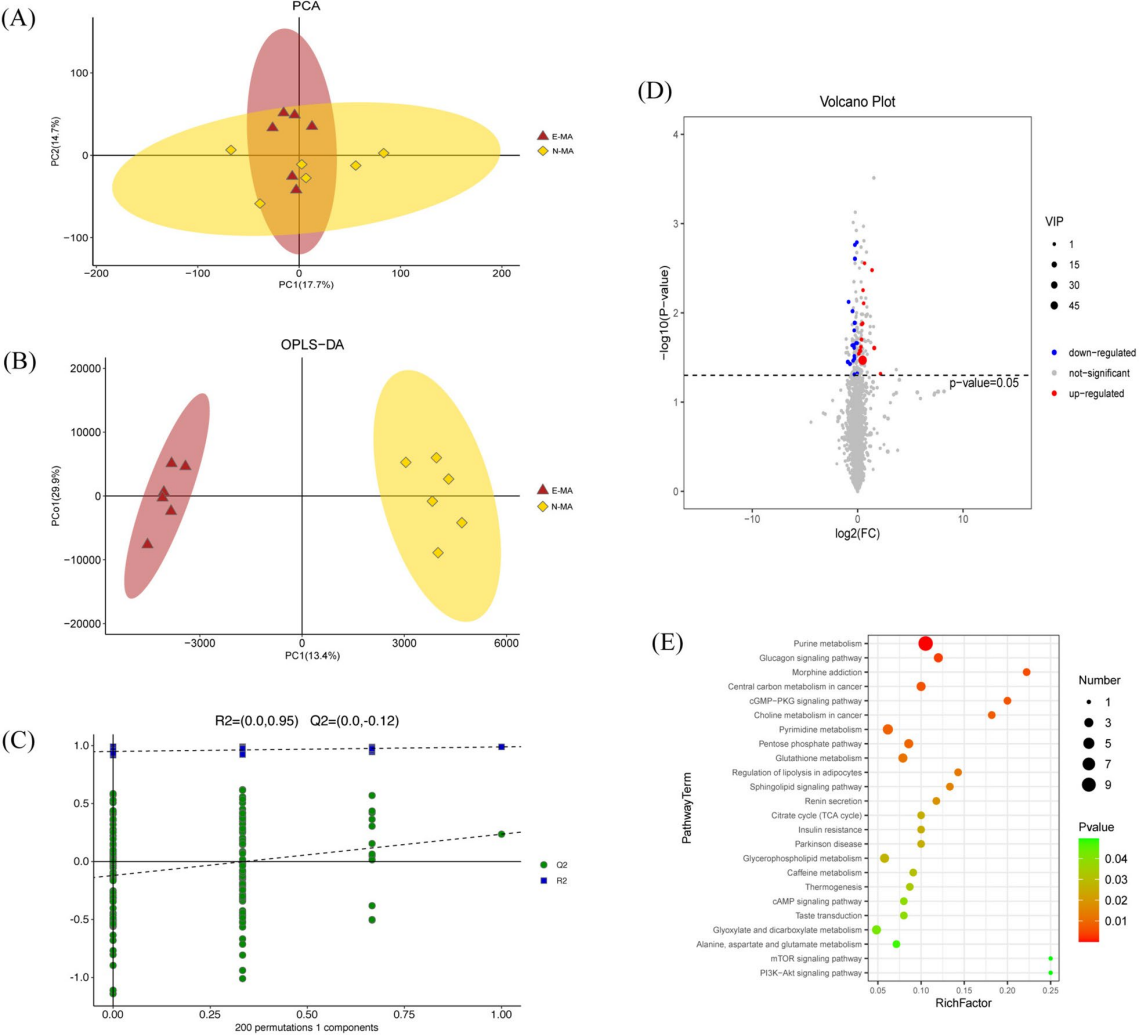
Differences in metabolic profiles between the E-MA and N-MA group. **A** PCA score plot. **B** OPLS-DA score plot. **C** 200- response permutation testing chart for OPLS-DA. **D** Volcano plots of differentially abundant metabolites. **E** Bubble chart of metabolic pathways affected by exercise from KEGG enrichment

Furthermore, the effect of aerobic exercise on MA-induced brain metabolic disorders was explored. The PCA plot is shown in Fig. 5A, and the metabolic differences between the E-MA and N-MA group were visualized in PCA2. The differences between the two groups can be explained clearly in OPLS-DA (Fig. 5B). In addition, the RPT results ofR2=(0.0, 0.95), Q2=(0.0, − 0.12) further ensure the reliability of this model (Fig. 5C). After ward, the thresholds were set to *p* < 0.05 and VIP > 1, and 32 differential metabolites were screened (Table 2). In comparison with the N-MA group, 13 metabolites were significantly increased in the E-MA group (red dots) and 19 metabolites were significantly decreased (blue dots, Fig. 5D). Finally, enrichment analysis (*P*<0.05) of the screened differential metabolites was carried out through the KEGG database, and five pathways were obtained (Fig. 5E), including purine metabolism, pentose phosphate pathway, glycosaminoglycan biosynthesis-heparan sulfate/heparin, choline metabolism in cancer, and glycosaminoglycan biosynthesis-chondroitin sulfate/dermatan sulfate.

**Table 2:** Differential metabolites screened from the E-MA and N-MA groups

NO.	Metabolite	*p* Value	VIP	Fold Change	Variations versus methamphetamine
1	PC(14:0/20:1(11Z))	0.034	68.468	1.389	↓
2	p-hydroxyfosinoprilat	0.023	4.175	0.769	↓
3	3-Methyl sulfolene	0.027	3.959	1.179	↑
4	PC(22:6(4Z,7Z,10Z,13Z,16Z,19Z)/0:0)	0.035	3.780	0.526	↓
5	Ribose 1-phosphate	0.013	3.729	0.824	↓
6	D-Glycerate 3-phosphate	0.025	3.632	2.979	↑
7	PI(20:4(5Z,8Z,11Z,14Z)/0:0)	0.002	3.593	0.837	↓
8	Dehydroepiandrosterone (DHEA)	0.010	2.806	0.714	↓
9	Adenosine 3’,5’-diphosphate	0.037	2.804	0.606	↓
10	4-[(Hydroxymethyl)nitrosoamino]-1-(3-pyridinyl)-1-butanone	0.032	2.300	0.811	↓
11	Asparaginyl-Valine	0.002	2.255	0.835	↓
12	Deoxyguanosine diphosphate (dGDP)	0.008	2.091	0.553	↓
13	(9R,13R)-1a,1b-dihomo-jasmonic acid	0.002	1.986	0.950	↓
14	L-Iduronate 2-sulfate	0.013	1.723	1.390	↑
15	Guanosine tetraphosphate adenosine	0.003	1.715	2.574	↑
16	Butyric acid	0.013	1.507	1.319	↑
17	Hydroxymethylphosphonate	0.034	1.498	0.745	↓
18	PC(24:0/P-18:1(11Z))	0.029	1.493	1.080	↑
19	Procyanidin B1	0.024	1.399	1.245	↑
20	Penciclovir	0.020	1.382	1.288	↑
21	PHODiA-PA	0.049	1.323	0.805	↓
22	3-Mercapto-2-methyl-1-butanol	0.016	1.300	0.796	↓
23	Xanthosine	0.006	1.243	1.429	↑
24	3,5-Dinitrosalicylic acid	0.022	1.203	0.972	↓
25	Dieporeticenin	0.023	1.198	0.699	↓
26	Linoleoyl ethanolamide	0.030	1.189	0.803	↓
27	6-Thiourate	0.048	1.163	0.975	↓
28	Grepafloxacin	0.008	1.071	1.484	↑
29	(Z)-15-Oxo-11-eicosenoic acid	0.022	1.058	0.906	↓
30	5’-CMP	0.025	1.046	0.795	↓
31	Tetraphyllin B sulfate	0.048	1.038	4.496	↑
32	1-Propenyl 1-(propylsulfinyl)propyl disulfide	0.003	1.018	1.580	↑

### Comparison of MA and exercise in metabolic changes

Six differential metabolites showing opposite trends under exercise and MA induction are listed in Table 3. Among them, the expression levels PC(22:6(4Z,7Z,10Z,13Z,16Z,19Z)/0:0), dehydroepiandrosterone (DHEA), PI(20:4(5Z,8Z,11Z,14Z)/0:0), p-hydroxyfosinoprilat, and PHODiA-PA in the N-MA group were increased, and the expression level of PC(24:0/P-18:1(11Z)) was lower thanthat of the C group. Exercise resulted in the opposite trend. Notably, the pathway analysis showed that these identified differential metabolites were mainly involved in glycerophospholipid metabolism, steroid hormone biosynthesis and degradation, and renin-angiotensin system (RAS) pathways. Moreover, eight differential metabolites had the same change trend under exercise and MA induction (Table 4). Both N-MA and E-MA decreased the expression of ribose 1-phosphate; 5’-CMP, adenosine 3’,5’-diphosphate, deoxyguanosine diphosphate (dGDP) and increased the expression of xanthosine, D-glycerate 3-phosphate, 3-methyl sulfolene, and penciclovir.

**Table 3:** Metabolites with different trends under E-MA and N-MA

NO.	Metabolite	Variations with E-MA	Variations with N-MA
1	PC(22:6(4Z,7Z,10Z,13Z,16Z,19Z)/0:0)	↓	↑
2	PI(20:4(5Z,8Z,11Z,14Z)/0:0)	↓	↑
3	p-hydroxyfosinoprilat	↓	↑
4	PHODiA-PA	↓	↑
5	Dehydroepiandrosterone (DHEA)	↓	↑
6	PC(24:0/P-18:1(11Z))	↑	↓

**Table 4:** Metabolites with the same trends under E-MA and N-MA

NO.	Metabolite	Variations with E-MA	Variations with N-MA
1	Ribose 1-phosphate	↓	↓
2	5’-CMP	↓	↓
3	Adenosine 3’,5’-diphosphate	↓	↓
4	Deoxyguanosine diphosphate (dGDP)	↓	↓
5	Xanthosine	↑	↑
6	D-Glycerate 3-phosphate	↑	↑
7	3-Methyl sulfolene	↑	↑
8	Penciclovir	↑	↑

## Discussion

In the present study, the MA-induced mice model of addiction was used, and the regulation of MA-dependent metabolic homeostasis in brain tissue was explored via aerobic exercise based on LC-MS. To our best knowledge, this study was the first to use LC-MS to investigate the effects of exercise on MA-dependent brain metabolism. The findings suggested that aerobic exercise can alter the expression of metabolites involved in glycerophospholipid metabolism, biosynthesis and metabolism of steroid hormones, and the RAS. The expression of PC(22:6(4Z,7Z,10Z,13Z,16Z,19Z)/0:0), DHEA, PI(20:4(5Z,8Z,11Z,14Z)/0:0), p-hydroxyfosinoprilat, PHODiA-PA, and PC(24:0/P-18:1(11Z)) were reversed after exercise intervention. Therefore, these six metabolites may serve as biomarkers for the exercise treatment of MA use.

Glycerophospholipid metabolites could serve as markers for the assessment of drug addiction [33, 51, 52], and the current findings demonstrated that this recommendation is equally applicable to MA addiction. Direct damage from MA exposure is associated with vascular toxicity [53], decreased BBB tight junctions and increased permeability [54], direct neurotoxic to neurons, and neuroinflammation [55]. The abnormal glycerophospholipid metabolism observed in this study may partially explain the occurrence of these injuries. Glycerophospholipids(GPLs) are the most common and abundant phospholipids in the body and the major component of membranes [56]. They play an important role in the dynamics of synaptic membranes and cooperate with synapsins to promote the exocytic and endocytosis of synaptic vesicles [57]. In addition, glycerophospholipid homeostasis has been recognized as a key factor in shaping neuronal morphology [58]. Glycerophospholipid metabolites are involved in regulating many biological processes as second messengers to ensure normal ion homeostasis in neurons and glial cells [59]. Notably, the dysregulated glycerophospholipid metabolism may lead to the adverse effect on nerve damage and inflammation [60]. Therefore, the disruption of BBB, central nervous system synaptic signaling by MA, and the development of MA-induced neuronal injury and inflammation may be caused by the disruption of glycerophospholipid metabolism.

Phosphatidylcholine (PC), phosphatidylinositol (PI), and phosphatidic acid (PA) are the major members of the glycerophospholipid family [61]. PC is the most abundant lipid class in the brain, and it participates in signaling during neuronal differentiation [62] and the recovery of neuronal differentiation under pathological conditions. Normally expressed PC has anti-inflammatory and antioxidant properties [62], But both abnormally high and low PC levels can affect the body’s energy metabolism and lead to disease development [63]. Furthermore, although PI is a small fraction of glycerophospholipids, it is extremely important for the nervous system [64]. PI participates in regulating synaptic function, including vesicle trafficking in the presynaptic and receptor modulation in the postsynaptic [65]. Moreover, PI and its phosphorylated forms play an important role in cell signaling and membrane trafficking [66]. PA is the precursor of all GPLs, an important component of GPL homeostasis during intracellular transport [67], and PA has received increasing attention as a second messenger and regulator of membrane shape [68]. In the present study, aerobic exercise can regulate the expression of the above three types of glycerophospholipids to normal levels. Therefore, the important role of exercise in maintaining BBB function, protecting neuronal function, reducing the inflammatory response and nerve damage, and improving synaptic plasticity may be carried out by regulating glycerophospholipid metabolic homeostasis [28, 69]. However, the detailed mechanism by which exercise affects drug addiction through abnormal glycerophospholipids metabolism has not been fully understood.

DHEA is defined as a “neurosteroid” when produced in the brain and acts as a modulator affecting inhibitory and excitatory neurotransmitters [70]. Endogenous DHEA levels in the brain are be positively correlated with drug abuse in studies involving addiction. Chronic cocaine administration leads to increased brain DHEA levels in animal models [71]. Based on clinical studies, DHEA levels in the serum of smokers are up-regulated compared with healthy populations [72]. The evidence supports the findings of this study that MA use leads to increased levels of DHEA in the brain. Notably, increased brain endogenous DHEA is reported as a compensatory protective mechanism to counteract cravings for substance abuse and promote recovery [67]. This phenomenon may be related to the activity of dopamine, sigma-1 receptors, glutamate receptors, and gamma-aminobutyric acid type A receptors [68]. MA administration can lead to cognitive impairment and decision-making impairment [69], which is one among the important reasons for the high relapse rate in MA. Overexpressed endogenous DHEA can inhibit hippocampal-based functions, which may affect the encoding and processing of space and cues [73]. In addition, DHEA levels are down-regulated after 6 weeks of substance withdrawal [72], which is consistent with the current findings that exercise tended to normalize endogenous DHEA levels in the brain. In conclusion, MA-induced elevation of DHEA levels in the brain appears to be a hallmark of brain injury, and exercise could modulate this condition.

The RAS in the brain exhibits pleiotropic properties and can be involved in neuroprotection and cognition, blood pressure regulation, stress, depression, alcohol addiction, and pain regulation [74]. RAS mainly exerts its important functions through the angiotensin-converting enzyme(ACE) [75]. *p*-Hydroxyfosinoprilat can inhibit the ACE activity [76]. When ACE is inhibited, the expression of the unconventional enkephalin Met-enkephalin-Arg-Phe is enhanced in the nucleus accumbens of mice, thus activating endogenous µ-opioid receptors and causing a cell type-specific long-term depression of glutamate release onto medium spiny projection neurons expressing the dopamine-1 receptor [77]. RAS may also be involved in maintaining BBB integrity in MA-addicted brains [78]. The observed MA-induced and elevated expression of p-hydroxyfosinoprilat in the mice brain may be a protective mechanism for resistance to brain injury and degradation of enkephalins by ACE. Moreover, physical exercise can adjust the expression of p-hydroxyfosinoprilat to normal levels, which may explain the mitigation of this damage, but the specific mechanism of this change is still unknown. Future studies are needed to explore the mechanisms of RAS in the treatment of MA-dependent patients.

### Limitations

The study had some limitations. First, our study was limited to young male mice and should be expanded to include differences related to gender, age. Second, we did not investigate differences in MA-induced brain metabolomics between different exercise protocols (e.g., acute exercise, resistance exercise, etc.). Furthermore, while changes in MA-induced brain metabolites and their exercise effects have been identified in this study, the exact molecular mechanism has not been further validated. This will also be the focus of our future work.

## Conclusions

To the best of our knowledge, this study was the first to use LC-MS technique to explore the effects of aerobic exercise on MA-induced brain metabolic profiles. This study elucidated the expression profile of MA-induced brain metabolites and demonstrated that aerobic exercise could modulate this change. In addition, differential metabolites affected by both MA and exercise were mainly enriched in glycerophospholipid metabolism, steroid hormone biosynthesis and degradation, and the RAS. These metabolites and pathways may play a key role in the treatment of MA use by exercise, worthy of attention. In conclusion, the findings of this study provide new theoretical support for exercise treatment of MA use.

### Supplementary Information

**Additional file 1.**

## Data Availability

The metabolomic datasets generated and analyzed during the current study are available in the ‘Baidu Netdisk’ (https://pan.baidu.com/s/1nPdoYfWtTXdCZxxTLyJYsA) with extraction code cdsu.
