# Supplementary material for: Effect of aerobic exercise on brain metabolite profiles in the mouse models of methamphetamine addiction: LC-MS-based metabolomics study
Source: BMC Psychiatry. 2023 Nov 17;23:852. doi: 10.1186/s12888-023-05351-1 (PMC10655403; doi:10.1186/s12888-023-05351-1)
Supplement: Supplementary file 1 — Additional file 1. [file 12888_2023_5351_MOESM1_ESM.pdf]

The metabolomics method

1 Materials and Reagents

Methanol, formic acid, water, and acetonitrile were purchased from Thermo Company. L-2-chlorophenylalanine was purchased from Shanghai Hengchuang Biotechnology Co., Ltd. All chemicals and solvents used in the study were of analytical or chromatographic grade.

2 Equipment

2.1. Automatic Sample Rapid Grinder (Wonbio-E, Shanghai Wanbai Biotechnology Co., Ltd.).

2.2 Ultrasonic cleaner. (F-060SD, Shenzhen Fuyang Technology Group Co.).

2.3 Tabletop high-speed frozen centrifuge (TGL-16MS, Shanghai Lu Xiang Yi Centrifuge Instrument Co.).

2.4 Freeze-concentrated centrifugal dryer (LNG-T98, Taicang Huamei Biochemical Instrument Factory).

2.5 High-Resolution Mass Spectrometer (QE plus, Thermo Fisher Scientific).

2.6 High-Performance Liquid Chromatograph (Dionex U3000 UHPLC, Thermo Fisher Scientific).

2.7 Chromatographic columns (ACQUITY UPLC HSS T3 (100 mm×2.1 mm, 1.8 um, Waters)).

3 Software

Progenesis QI v2.3.

4 Procedure

4.1 Pre-processing

4.1.1 Precisely weigh 30 mg of tissue sample into a 1.5 mL EP tube, add 20 μL of internal standard (L-2-chlorophenylalanine, 0.06 mg/mL; methanol configuration) and 400 μL of methanol-water (V:V=4:1).

4.1.2 Add two small steel balls, pre-chill them in a refrigerator at -20°C for 2 min and put them in a grinder (60 Hz, 2 min).

4.1.3 Extracted by ultrasonication in an ice-water bath for 10 min and left to stand at -20 ℃ for 30 min.

4.1.4 Centrifuge for 10 min (13000 rpm, 4°C) and evaporate 300 μL of supernatant into the LC-MS injection vial.

4.1.5 Resolubilization with 300 μL of methanol-water (V: V = 1:4) (vortex for 30 s, sonication for min 3).

4.1.6 Standing at －20 °C for 2 hours.

4.1.7 Centrifuge for 10 min (13000 rpm, 4 °C), aspirate 150 μL of supernatant with a syringe, filter using a 0.22 μm organic phase pinhole filter, transfer to an LC injection vial, and store at -80 °C until LC-MS analysis is performed.

4.1.8 Quality control samples (QC) were prepared by mixing equal volumes of extracts from all samples.

Note: All extraction reagents were pre-cooled at -20 ℃ before use.

4.2. Liquid chromatography-mass spectrometry analysis conditions

4.2.1 Chromatographic conditions

Chromatographic columns: ACQUITY UPLC HSS T3 (100 mm×2.1 mm, 1.8 um).

Column temperature: 45 ℃.

Mobile phases: A-water (containing 0.1% formic acid), B-acetonitrile (containing 0.1% formic acid).

Flow Rate: 0.35 mL/min.

Injection volume: 2 μL.

Elution gradient

Time	A%	B%
0	95	5
2	95	5
4	70	30
8	50	50
10	20	80

14	0	100
15	0	100
15.1	95	5
16	95	5

4.2.2 Mass Spectrometry Conditions

Sample mass spectrometry signal acquisition using positive and negative ion scan mode respectively.

Mass Spectrometry Parameters

Parameters	Positive ions	Negative ions
Spray Voltage (V)	3800	- 3000
Capillary Temperature (°C)	320	320
Aux gas heater temperature (°C)	350	350
Sheath Gas Flow Rate (Arb)	35	35
Aux gas flow rate (Arb)	8	8
S-lens RF level	50	50
Mass range (m/z)	100-1200	100-1200
Full ms resolution	70000	70000
MS/MS resolution	17500	17500
NCE/stepped NCE	10, 20, 40	10, 20, 40

5 Data analysis

5.1 Pre-processing

Raw data were processed by metabolomics software Progenesis QI v2.3 software (Nonlinear Dynamics, Newcastle, UK) for baseline filtering, peak identification, integration, retention time correction, peak alignment and normalization with the following key parameters.

precursor tolerance: 5 ppm / 10ppm (self-built library),

product tolerance: 10 ppm / 20ppm (self-built library),

product ion threshold: 5%.

Compounds were characterized based on exact mass numbers, secondary fragmentation, and isotopic distribution using The Human Metabolome Database (HMDB), Lipidmaps (v2.3), and METLIN databases as well as self-built libraries. For the extracted data, the ion peaks with missing values (0 value) >50% within the group were deleted and the 0 value was replaced by half of the minimum value, and the compounds obtained from the characterization were screened according to the compound characterization results in scoring (Score), with a screening criterion of 36 out of 60 points, and below 36 points were considered as inaccurate and deleted. Finally, the positive and negative ion data were combined into a data matrix table, which contains all the information extracted from the original data that can be used for analysis, and the subsequent investigation was based on this, and the substance peaks and metabolite statistics were tabulated as follows.

	ALL	Negative	Positive
Number of material peaks	8864	3837	5027
Number of metabolites	2793	763	2030

5.2 Statistical Analysis

We first used multivariate statistical analysis for data processing. Unsupervised principal component analysis (PCA) was first used to observe the overall distribution among the samples and the stability of the whole analysis process, and then supervised orthogonal partial least squares analysis (OPLS-DA) was used to distinguish the overall differences in metabolic profiles among the groups and find the differential metabolites between the groups. To prevent model overfitting, 7-fold cross-validation, and 200 times response permutation testing, RPT was used to examine the quality of the model. We then used Student's t-test and Fold change analysis for descriptive and statistical inference. A combination of multidimensional and unidimensional analyses was used to screen for differential metabolites between groups. In OPLS-DA analysis, Variable important in projection (VIP) can be used to measure the intensity and explanatory power of the expression pattern of each metabolite on the categorical discrimination of each group of samples, to mine for biologically significant differential metabolites, and further to verify whether the differential metabolites between groups are significant using t-test. The screening criteria were VIP value > 1 for the first principal component of the OPLS-DA model and p-value value < 0.05 for the t-test. Finally,

we performed pathway enrichment analysis using the KEGG ID of differential metabolites to obtain metabolic pathway enrichment results. Hypergeometric tests were applied to identify pathway entries that were significantly enriched in significantly differentially expressed metabolites compared to the whole background. The p-value$\leq$0.05 was taken as the queue value, and the pathway satisfying this condition was the pathway significantly enriched in the differential metabolites. the smaller the p-value, the more significant the differential of this metabolic pathway.
